# The Anatomical Basis of Heavy Metal Responses in Legumes and Their Impact on Plant–Rhizosphere Interactions

**DOI:** 10.3390/plants11192554

**Published:** 2022-09-28

**Authors:** Arun K. Pandey, Lana Zorić, Ting Sun, Dunja Karanović, Pingping Fang, Milan Borišev, Xinyang Wu, Jadranka Luković, Pei Xu

**Affiliations:** 1 College of Life Sciences, China Jiliang University, Hangzhou 310018, China; 2 Department of Biology and Ecology, Faculty of Sciences, University of Novi Sad, 21121 Novi Sad, Serbia; 3 Key Laboratory of Specialty Agri-Product Quality and Hazard Controlling Technology of Zhejiang Province, Hangzhou 310018, China

**Keywords:** heavy metals, legumes, anatomy, phytoremediation, translocation, rhizosphere

## Abstract

Rapid industrialization, urbanization, and mine tailings runoff are the main sources of heavy metal contamination of agricultural land, which has become one of the major constraints to crop growth and productivity. Finding appropriate solutions to protect plants and agricultural land from heavy metal pollution/harmful effects is important for sustainable development. Phytoremediation and plant growth-promoting rhizobacteria (PGPR) are promising methods for this purpose, which both heavily rely on an appropriate understanding of the anatomical structure of plants. Specialized anatomical features, such as those of epidermis and endodermis and changes in the root vascular tissue, are often associated with heavy metal tolerance in legumes. This review emphasizes the uptake and transport of heavy metals by legume plants that can be used to enhance soil detoxification by phytoremediation processes. Moreover, the review also focuses on the role of rhizospheric organisms in the facilitation of heavy metal uptake, the various mechanisms of enhancing the availability of heavy metals in the rhizosphere, the genetic diversity, and the microbial genera involved in these processes. The information presented here can be exploited for improving the growth and productivity of legume plants in metal-prone soils.

## 1. Introduction

Plants, as sessile organisms, are exposed to various challenging environmental conditions throughout their lifecycle that adversely affect their growth and developmental processes [1,2]. Heavy metal toxicity reduces plant growth/productivity and causes severe health hazards in humans. Various metals and metalloids, such as arsenic (As), cadmium (Cd), mercury (Hg), lead (Pb), nickel (Ni), zinc (Zn), cobalt (Co), aluminum (Al), and chromium (Cr), induce severe toxicity when they enter into the soil agro-ecosystem either through natural processes or by anthropogenic activities [3,4]. Natural sources of heavy metals (HM) contamination include weathering of rocks, soil erosion, burning of forests, and volcanic eruptions, whereas anthropogenic sources involve extensive mining, metal smelting, application of chemical fertilizers, industrial/sewage discharge, and coal combustion [5,6]. Rapid industrialization and technological advancements have altered the normal geochemical cycle of metals/elements, which in turn have accelerated their increment in soil horizons [7,8]. Increased bioaccumulation of heavy metals beyond the threshold level has shown a negative impact on the natural food chain and microbial flora and is therefore now being perceived as an imminent threat to the ecosystem and environment [6,9].

In plants, heavy metals are absorbed by the roots and transported to shoots, causing significant damage to root and shoot cells, and internal organelles, such as chloroplast and mitochondria, thus reducing energy production and imposing oxidative stress, and ultimately affecting plant morphology and survival rate [10,11]. While many plants are sensitive to heavy metals, some species are tolerant to them or are even hyper-accumulators [12]. Phytoremediation is a branch of bioremediation that uses plants for the removal of pollutants from contaminated soils [13,14]. It is effective for contaminated sites with pollutants that are distributed within the root zone of the plants [11]. The rhizosphere bacteria that inhabit the root zone of the plants play an important role in the phytoremediation process, via various mechanisms. Most of the physical and chemical activities taking place in the rhizosphere have a direct impact on the root system. It is well understood that plant–microbe interaction determines the efficiency of metal extraction. Whiting et al. [15] showed that Thlaspi caerulescens plants inoculated with rhizosphere bacteria accumulated high amounts of Zn. Different mechanisms, such as exopolysaccharide (EPS) production, rhizosphere acidification through organic acids, siderophore production, indole-3-acetic (IAA), or 1-amino1-cyclopropanoic acid (ACC) deaminase production, and the release of growth-limiting nutrients from the soil are involved in improving the rate of heavy metal accumulation in plants [11].

Family Fabaceae comprises more than 19,000 species, including many agriculturally important crops, such as soybean, pea, and common bean, which are essential for global food security [16]. Besides staple legume crops, underutilized legumes, such as cowpea and rice bean, have significantly contributed towards the dietary requirements of the rural areas, particularly under adverse environmental conditions, such as drought and famine situations [17,18]. These crops are the life-savers for millions of people where assuring food and nutritional security is one of the major challenges, particularly in ancestral subsistence farming activities [19]. Although many legume cultivation areas are facing HM contaminations, to date limited information is available regarding the responsive and adaptive mechanisms in legume crops to heavy metal exposures in comparison to other major crops. Therefore, the present review is an attempt to evaluate the recent discoveries and breakthroughs in deciphering various mechanisms/strategies that are used by legume plants to respond to heavy metal stress. Specifically, this review highlights the anatomical basis associated with heavy metal uptake and translocation, which is rare among the abundant literature, as well as the involvement of the symbiotic rhizosphere microbes, a characteristic of legume plants, in this process. This collective information will be valuable for guiding the improvement of the growth and productivity of legume plants under metal-prone soils.

## 2. The Adverse Impacts of Heavy Metals on Legumes

Heavy metals at low concentrations can play a stimulative role in plants’ growth and development [5,20]; however, when their levels reach beyond a certain threshold, they act as an imminent threat to plants [21]. The general effects of various metals in a plant are given in Table 1. One of the most important effects of Cd stress on legume plants is growth inhibition. Root and shoot dry weight and length were significantly reduced in *Glycine max* (L.) under Cd stress [22], which is usually a consequence of reduced photosynthetic rate and disturbance in uptake and distribution of macro-and micro-nutrients. Cd stress also significantly decreased growth and yield parameters of soybean plants, e.g., plant height, number of branches and leaves, total leaf area, shoot dry weight, number of pods and seeds/plant, seed yield, and weight. All these parameters were negatively correlated with Cd concentration in the soil [23]. In runner bean (*Phaseouls coccineus* L.), Cd-induced a reduction of seedling leaf area to 39%, whilst Cd applied in later growth stages showed a smoother effect [24]. Seed germination of *Phaseolus vulgaris* was lowered by 68–98%, depending on the applied Cd concentration [25]. Zornoza et al. [26] found the reduction of shoot and root dry weight in white lupin treated with Cd by 38% and 15%, respectively, which was owing to the reduced internodal length, plant height, and lateral root development. Growth reduction by Cd is largely attributed to disturbed water and mineral nutrients uptake, which is often associated with decreased shoot water content [27]. Growth reduction in Cd and Pb treated *Trigonella foenum graecum* L. resulted in smaller vegetative organs, lower dry weight, smaller leaf area, and number of leaves and branches [28]. High concentrations of Fe reduced root and shoot biomass in peanut [29]. It is also reported that Al caused a reduction in alfalfa root length, weight, and activity. Number of leaves, total leaf area, and leaf dry mass declined significantly in common bean (*Phaseolus vulgaris* L.) plants under Zn treatment [30]. Macroscopic alterations induced by As in soybean included a decreased number of lateral roots, abnormal thickening and darkening of root, necrotic, and slimy main root apex [31]. The roots of *Vicia villosa* treated with As showed a decrease in root length, which resulted in lower uptake and transport of water and nutrients, and consequently decreased growth of aerial plant parts [32]. In addition to length reduction, root color of treated *Vicia villosa* plants were brownish and darker. Root growth reduction, which was particularly prominent in lateral roots, as well as abnormal development of the root cap, occurred in *Cajanus cajani* plants treated with As [33].

On the other hand, some legumes particularly soybean, common bean, and hyacinth bean (*Lablab purpureus* (L.) are most commonly used for phytoextraction/phytostabilization mainly due to their ability to colonize metal-enriched soils to restore their fertility thereby stimulating crop growth and productivity [34]. The Indian bean, sometimes known as the hyacinth bean, has also been reported to be tolerant to a number of heavy metals, including Cd, Hg, Pb, Zn, P, and Cr [35,36]. Thanks to the early availability of whole-genome sequence and well-established genetics, *Medicago* has been thoroughly investigated for its phytoremediation [37]. It is reported that *Medicago sativa* with *Sinorhizobium* (syn. Ensifer) *meliloti* and *Sinorhizobium medica* enhanced nodulation efficiency in *Medicago* plants, resulting in increased metal bioaccumulation through root nodules, which promotes land restoration and phytostabilization [37,38]. Likewise, several other studies reported that by using *Lens culinaris*, *Lupinus luteus, Sulla coronaria, Vicia faba*, and *Lablab purpureus* with *Pseudomonas* sp. *Az13, Bradyrhizobium* sp. *750* and *Ochrobactrum cytisi Azn6.2* significantly improved plant grfowth and yield, even though they accumulated more heavy metals than non-inoculated plants [39,40]. Indeed, the bioaccumulation of heavy metals in food crops and their effects on human health are of great concern worldwide. The concentration of heavy metals in the fruit of *Pisum sativum* L. (garden pea) grown in contaminated soils in comparison to non-polluted soils, and their adverse effects on plants are mentioned in Table 1. Nevertheless, more collective efforts are necessary to enhance legume-based phytoremediation by reprogramming the host–plant symbiotic relationship through biotechnological interventions to increase metal tolerance and phytoremediation ability in plants.

## 3. Anatomical Basis Related to HM Uptake and Translocation in Legumes

**Figure 1:**
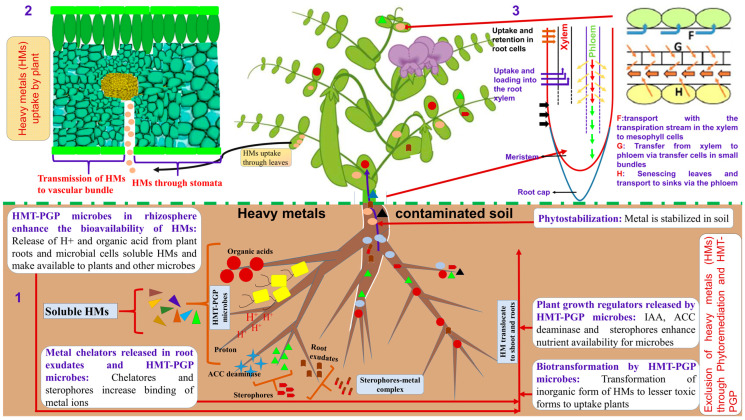
Schematic representation of the mechanisms involved in plant uptake, transportation, and phytoremediation of HMs: (1) Heavy metal tolerant-plant growth-promoting (HMT-PGP) microbes in ensuring plant survival and growth in contaminated soils; (2) the processes involved in the distribution and redistribution of heavy metals in plants; and (3) and remobilization from senescing leaves and transport to sinks via the phloem.

Heavy metal uptake and transport in plants depend on many factors, such as the element characteristics, soil type, plant species, and plant organ. Among them, the anatomical characteristics of the plant organs exposed to heavy metal stress are more often neglected, despite its importance in affecting the metal uptake and transport [49]. For example, the inhibition of root growth in Cd-stressed plants is attributed to the aberrations in apical meristematic tissue, reduction or inhibition of mitosis, and reduced production of new cells, as demonstrated for pea (*Pisum sativum* L.) plants [50]. Kazemi et al. [51] reported that the cadmium ions disrupt mitosis in chickpea plants and also disturb root and stem growth. Such anatomical and morphological changes reduce water and mineral uptake by roots, thereby compromising the plant growth rate potential [22]. In white lupin (*Lupinus albus* L.), Mn and Ni are transferred from root to shoot, allowing significant amounts of Mn to reach the subsequently emerging leaves. On the other hand, the transfer factor values exceeding 1 recorded for Cd, Zn, and Cu in mungbean (*Vigna radiata* (L.) Wilczek (on average 1.1, 3.0 and 1.4, respectively) and Zn in chickpea (*Cicer arientinum* L.) (approximately 2.0) suggest a higher concentration of these metals in shoots compared to roots in these species [52]. Knowledge of the anatomical basis for metal uptake and transport, as well as the consequent plant adaptations to metal stress, is therefore fundamental for understanding plant–environment interactions in changing environments and is required for enhancing the management and breeding of superior crop cultivars [53]. In the following paragraphs, we detail the anatomical basis related to HM uptake and translocation in various legume organs (Figure 1).

### 3.1. Root

The root is the first organ in contact with soil and is thus responsible for the uptake and translocation of water and ions. Root structure strongly affects these processes, whereby root apex and young parts of the root actively absorb water and inorganic substances, together with heavy metal ions, through rhizodermis [54]. Many plants develop several anatomical alterations to strengthen its structures and restrict the metal uptake. The most common changes include cell wall modifications and impregnation by secondary metabolites, especially in peripheral tissues (rhizodermis, exodermis, and endodermis), which are in direct contact with the pollutants [53]. Some ions are absorbed to cell walls, thus becoming less toxic and contributing to metal retention and elimination from further transport and processes. The formation of apoplastic barriers, such as Casparian bands and suberin lamellae, in exo- and endodermis, usually closer to the root tip, is a further adaptation aimed at limiting metal uptake and transfer to the vascular cylinder [54]. In general, a decrease in cell division and cell size (due to reduced growth and lower elasticity of cell walls), along with a decrease in vessel size (and thus reduced vascular cylinder area), contribute jointly to a reduction in root diameter in plants under heavy metal stress [55].

Moreover, findings reported by Römer et al. [56] indicate that significant proportion of Cd does not pass the Casparian strip of the lupin roots, but stays adsorbed in the root apoplast. The authors attribute this phenomenon to apoplastic Cd fraction, resulting in only 6% of Cd being translocated to the shoot, compared to 30% in *Lupinus angustifolius* L. These observations were confirmed by Zornoza et al. [26] who noted that strong (88%) retention of Cd in the white lupin roots is due to its binding to the cell walls as a defense mechanism. In the sample analyzed by Page et al. [57], more than 80% of Cd and Co within the white lupin root was present in the cortex, and more than 80% of Ni was stored in the vascular cylinder, whereas Mn and Zn were almost equally distributed between the cortex and the vascular cylinder. Poor transfer of metals into vascular cylinder limits the transfer to the shoot, thus providing a defense mechanism against their toxicity. Cell walls (mainly of endodermis, phloem, and xylem) were also the main site of Cu accumulation in common bean [58].

Soil metal contamination exhibits an immediate effect on rhizodermis, inducing its disintegration and damage of rhizodermal cells, as well as changes in root hair number and size [53]. As an increased root hair number could be an adaptive strategy to maintain water and mineral uptake, their reduced number or absence could be an indicator of high metal toxicity. Rahoui et al. [59] noted that root hairs in Cd (100 µM)-treated *Medicago truncatula* Gaert became more numerous and condensed but inflated and deformed. Talukdar [60] reported the absence of root hairs and loosening of vascular bundles, cortex, and pith regions of bean roots under As (50 µM) stress. More recently, Ibañez et al. [32] observed reduced turgidity and thickening of the rhizodermal and subrhizodermal parenchyma layers in the roots of *Vicia villosa* treated with As (5–50 µM), which were accompanied by dark deposits.

Although an increased cortex thickness could provide resistance to radial water flow and thus reduce heavy metal transport, the disintegration of root cortical cells is a more prevalent phenomenon [53]. Liza et al. [61] recorded a decrease in the root diameter of chickpea plants exposed to Cd (250–1000 µM) due to a decreased number of layers of cortical cells (from 12–14 in control plants, to 8–10 in Cd-treated plants). Conversely, root diameter was shown by Perez Chaca et al. [22] to increase in Cd (40 µM) treated soybean plants, as a result of an increase in the cortex area and the size of cortical parenchyma cells. In the study conducted by El Hocine et al. [25], although no significant changes in the root diameter of common bean plants exposed to Cd (0.25–1 gL^−1^) were noted, cortex zone enlargement and rhizodermis rupture at some parts of the root were noted. Fusconi et al. [50] reported an increased size of cortical parenchyma cells in pea roots under low Cd stress (2.5 µM), which decreased with increasing Cd concentration (25 and 250 µM). More recently, Gzyl et al. [62] observed plasmolysis in some cortical cells of Cd (170 µM) treated soybean seedlings, along with subcellular changes, such as vacuolization, presence of autophagic bodies and deposits in vacuoles, changes in nucleolus structure and callose deposition, which indicated an active root cell metabolism aimed at sustaining cell functioning. According to the analyses conducted by Ahmad et al. [28], the proportion of cortex decreased in Cd (0–50 µg/g of soil) and Pb (0–200 µg/g of soil)-treated *Trigonella foenum graecum* L., while Pb treatment also induced cell shape distortion. However, in Al-stressed (100 µM) alfalfa roots, Wang et al. [63] noted enlarged cortex, as a result of cell enlargement, which directly caused swelling of the root tip. The authors attributed cell enlargement to vacuole expansion, which allows plants to store excessive amounts of Al but might compromise root functioning and growth. Lavres et al. [64] observed that high Mn concentration (200 µM) led to soybean root thickening, without inducing alterations in the cells of dermal tissues, exodermis, and endodermis. The authors posited that root thickening may have been caused by suberization and lignification of cortex cells, resulting in reduced water and nutrient uptake. They further argued that the maintenance of the cell integrity improves root compartmentalization of Mn, thereby reducing its transport from roots to shoots. According to Lavres et al. [64], intercellular spaces may contribute to a greater accumulation of Mn in the roots, providing greater tolerance to excess Mn by inhibiting its transport to shoot. Shukry and Al-Osaimi [30] found that increasing Zn concentrations (1, 200, 600, and 1200 mM) cause an increase in bean root thickness, cortex, and vascular bundle width. A similar conclusion was made by dos Santos et al. [65], who found that soybean plants exposed to high Zn concentration (2000 µM) had thicker rhizodermis, endodermis, cortex, vascular cylinder, and metaxylem. In the study conducted by Armendariz et al. [31], As (25–200 µM)-treated soybean roots were characterized by broken cells in the outer layer, a reduction in the cortex area (due to decreased cell size but not the number of cell layers) and dark deposits in cortex cells. Pandey and Bhatt [66] reported that As (200–1000 mgL^−1^) exposure caused structural alterations in mungbean roots, such as changes in the size, shape, color, and arrangement of cortical parenchyma cells, breakdown of cortex and endodermal cells, damaged vascular bundle, and distorted pericycle. In an earlier study, Sresty and Rao [67] noted that Ni (0.5 and 1.5 mM) and Zn (2.5 and 7.5 mM) toxicity cause disintegration of cell organelles, disruption of membranes, condensation of chromatin material and increase in the number of nucleoli in the main root of pigeon pea.

Exo- and endodermis, with their cell walls incrusted with lipophilic and aromatic substances, lignin and suberin, have a barrier function in the radial transport of metals, thus protecting the plant from heavy metal stress [53,68]. Deformation and thickening of their cell walls, suberization or lignification led to the obstruction of metal entry into the vascular cylinder. Exposure of chickpea roots to 62 µg of Cd was found by Kazemi et al. [51] to result in the accumulation of suberine not only in radial but also in tangential cell walls of endodermal cells. However, at higher Cd concentrations (125 and 250 µg), the suberine level decreased, whereas the number of root cortex layers was unaffected by Cd exposure. In a more recent study, Liza et al. [61] noted that Cd (250–1000 µM)-treated roots of chickpea endodermal cells were smaller and thicker-walled compared to control plants.

Heavy metal entry into vascular tissue and transport to aboveground organs can also be inhibited through the anatomical changes in xylem, mainly in xylogenesis, the disintegration of xylem, vessel characteristics, and depositions [53]. In chickpea roots exposed to Cd (125 µg), Kazemi et al. [51] observed rearrangement of vessels, whereby further increase of Cd concentration (250 µg) led to a reduction in the lignification of vessel cell walls and the number of xylem elements. Moreover, Liza et al. [61] noted that the diameter of metaxylem vessels declined in Cd (250–1000 µM)-treated roots of chickpea plants. Cd (0.25–1 gL^−1^) was also found to reduce cell division and differentiation in the vascular cylinder of bean roots, which resulted in a smaller number and size of vessels [25]. In contrast, Pérez Chaca et al. [22] observed significant hypertrophy in the phloem and metaxylem elements of soybean plants under Cd (40 µM) stress, which led to the enlargement of the vascular cylinder area. The author further reported premature division of pericycle cells, while noting that protoxylem elements had differently thickened cell walls and dark intracellular content, which was tentatively attributed to metal retention by the walls. Gzyl et al. [62] also reported a more advanced xylem formation process in roots of Cd (85 and 170 µM) treated soybean seedlings. In the study conducted by Rahoui et al. [59], lignification in xylem increased, together with cellulose and pectin deposits in xylem and phloem of Cd (100 µM) treated roots of *M. truncatula*, already near the root tip. According to Ahmad et al. [28], in Cd (0–50 µg/g of soil) and Pb (0–200 µg/g of soil) treated *T. foenum graecum*, the proportion of vascular tissue increased, whereas the vessel density and size and xylem fiber length significantly declined. Exposure to Al (100 µM) was also found to reduce stele diameter in alfalfa roots by Wang et al. [63]. Available evidence further suggests that Ni (0.1 µmol) reduces the phloem and xylem diameter in soybean roots [69], while excess Mn (0.5 µmol) causes disorganization of xylem vessels [64]. However, no alteration or external cell derangement of the soybean root was observed by dos Santos et al. [65] in the presence of Mn (10–300 µM), while rhizodermis, phloem, and xylem diameter were significantly reduced. Dark depositions had been previously recorded in the vascular cylinder, within xylem vessel elements and phloem cell walls of soybean roots treated with As (25–200 µM), which could be considered a plant adaptation aimed at preventing As translocation to the aboveground tissues [31]. Findings reported by Ibañez et al. [32] indicate that root diameter, cortex length, and vascular cylinder size in *V. villosa* roots treated with As (V) (25 µM) were reduced, but no significant changes were noted in samples treated with As (III) at the same concentration. Pita-Barbosa et al. [33] recorded an increase in the vascular cylinder/root diameter ratio in *Cajanus cajan* (L.) DC roots treated with As (1.5 mg L^−1^), accompanied by a reduction in secondary xylem vessel diameter due to the negative effect of arsenic on cambium activity.

### 3.2. Stem

As heavy metals enter into the stem from the root via vascular tissue, mainly xylem, vasculature and the surrounding tissues are the main sites of anatomical changes [53]. Liza et al. [61] noted that stem diameter decreased in chickpea plants under Cd (250–1000 µM) exposure, predominantly due to the reduction in the size of cells and vascular elements. Stem epidermal cells were thicker and with a greater number of multicellular glandular trichomes compared to the control plants, suggesting that this is a defense mechanism in response to stress. The authors further observed that, at higher Cd concentrations (1000 µM), trichomes were higher in length and their heads were damaged, while stem cortex was less developed, with 10–12 cortical cell layers, compared to 14–16 in control plants. The cambium ring was thin and produced a smaller number of smaller-sized vessels, which contained phenolic compounds under higher Cd concentrations. The number of sclerenchymatous groups above the phloem also increased. In the study of El Hocine et al. [25], the stem diameter of common bean plants was not significantly affected by Cd (0.25–1 gL^−1^) exposure, but evidence of stem cortex enlargement was noted, along with a decreased number and size of xylem vessels. Ahmad et al. [28] reported an increased proportion of cortex, and a significant decrease in vessel density and size, as well as xylem fiber length in Cd (0–50 µg/g of soil) and Pb (0–200 µg/g of soil)-treated *T. foenum graecum*.

Increased Zn concentrations (1, 200, 600 and 1200 mM) have been found to decrease stem cortex width of common bean plants stepwise, as well as the number of cortical cell rows and vascular bundles [30]. According to Pandey and Bhatt [66], As (200–1000 mg L^−1^)-treated mungbean stems exhibited distorted epidermis, blackening of cortex cell walls, large pith, decreased and damaged vascular bundles, as well as decreased intercellular spaces. Conversely, Talukdar [60] recorded intact vascular bundle and cortex in stems, leaves, and petioles of As (50 µM) treated bean plants, whereas Tripathi et al. [70] noted that stem cortex region in chickpea was most severely affected by As (100 mg kg^−1^) stress. These authors also reported a reduction in the number of collenchyma layers from 4–5 to 2–3, deformation and loose arrangement of collenchyma cells, deformations of sclerenchymatous cells above phloem, as well as reduced trichome turgidity and density.

### 3.3. Leaf

The defensive strategy adopted by most plants involves limiting metal translocation to leaves and consequently protecting photosynthetic tissues [53]. Although metals are translocated to leaves in relatively limited quantities, even small doses can cause severe anatomical changes in the leaf, primarily a decreased size of leaf cells, vessels, and vascular bundles, which may affect stomatal parameters and synthesis of pigments [55].

Cd induces changes in the photosynthetic pigment concentrations, as well as in the structure of photosynthetic tissue, thus affecting the photosynthesis process. Chlorophyll degradation, inhibition of its biosynthesis and reduced photosynthesis rate have been found to occur in plants exposed to Cd stress. Cd also competes with other divalent cations, including Mg, as one of the constituents of the chlorophyll molecule [26,29]. Cd exposure (50–200 ppm and 40 µM) was found by Abdo et al. [23] and Perez Chaca et al. [32] to decrease the content of chlorophyll a, b and carotenoids, as well as reduce the number of chloroplasts in soybean leaves. Similar results were obtained for peanut (10–100 µM) and common bean (0.25–1 g L^−1^) [25,71]. In an earlier study, Skórzynska-Polit et al. [24] observed that the chloroplasts of older leaves of Cd (0.25 µM)- treated runner bean plants were smaller, of poorly differentiated ultrastructure and with a lower number of internal thylakoids, which resulted in partial degeneration of internal thylakoids and more numerous plastoglobuli. A reduction in total chlorophyll content was observed in alfalfa leaves following Al (100 µM) exposure [63] as well as in soybean leaves treated with As (25–200 µM) [27]. Kazemi et al. [51] recorded alterations in the photosynthetic process of chickpea plants induced by Cd (62–250 µg/g) due to, among other factors, structural changes, suppressed chlorophyll formation, decreased pigment concentration, and stomatal closure. Elevated Cd (10–100 µM), Zn (200–1000 µM), and Fe (10–100 µM) content were found to inhibit net photosynthetic and transpiration rate and stomatal conductance in peanut leaves [29,71,72].

Most stomata close in response to metal exposure, which, together with their decreased size and density, exerts a negative effect on transpiration, photosynthesis, and gas exchange [55]. Shi et al. and Cai et al. [71,72] found that the adaxial epidermis thickness and stomatal density in peanut leaves increased, whereas stomata size decreased due to Cd (10–100 µM) and Zn (200–1000 µM) treatment. However, in a subsequent investigation, Shi et al. [29] failed to find a link between Cd (0.2 µM) treatment and stomatal density. The analyses conducted by Ahmad et al. [28], however, revealed a decline in trichome density, stomatal density and size, along with an increase in trichome length in Cd (0–50 µg/g of soil) and Pb (0–200 µg/g of soil) treated leaves of *T. foenum graecum*. Dos Reis et al. [69] found that the epidermal thickness of soybean leaves decreases under Ni (0.1 µmol) stress, thereby compromising plant’s resistance to mechanical, chemical and biological damage. Ni also decreased CO_2_ assimilation rate, stomatal conductance, and transpiration, which resulted in a lower biomass of soybean plants. These effects were also associated with As (25 µM) [73] and Mn (2–300 µmol L^−1^) exposure [65], although these metals induced hypertrophy of the leaf adaxial epidermis and the formation of necrotic spots. In the study of Gupta and Bhatnagar [74], exposure of mungbean to As (5–35 mg kg^−1^) resulted in reduced stomata size, permanently closed stomata aperture, and thickened periclinal walls of the guard cells, while the frequency of fused and abnormal stomata increased. The authors further noted that As caused disintegration of cytoplasm and organelles of guard cells and affected microtubule arrangement, and consequently cell division and differentiation, resulting in various stomata abnormalities, confirming its cytotoxic effect. Trichome density on mungbean leaves decreased as the As concentration increased from 5 to 35 mg kg^−1^ whereby trichomes became smaller in size and displayed structural disruptions. Energy dispersive x-ray microanalysis further revealed that the highest concentrations of As occurred in the non-photosynthetic tissues, epidermal cells, and trichomes, while no As was detected in mesophyll tissue. This pattern might be associated with a detoxification mechanism, whereby the trichomes act as a sink for excessive As.

Mesophyll thickness has been found to decrease in soybean plants under Cd (40 µM) stress, due to the decreased size of palisade cells and consequently palisade tissue thickness [22]. According to Abdo et al. [23], in soybean plants treated with 100 ppm of Cd, lamina and midvein thickness decreased by 8.6% and 37.9%, respectively, due to the reduction in the palisade and spongy tissue thickness, as well as in the dimensions of main vein bundle components. The authors further noted that the number of xylem rows and vessels per midvein bundle and vessel diameter decreased by 27.3, 30.6, and 28.6%, respectively. On the other hand, in the study conducted by Shi et al. and Cai et al. [71,72], peanut leaves developed abundant palisade tissue under both Cd (10–100 µM) and Zn (200–1000 µM) treatments and consequently increased lamina thickness and palisade/spongy tissue ratio. Similar findings were reported by Shi et al. [29], who observed an increase in spongy tissue thickness, while presence of Fe (10–100 µM) did not significantly affect peanut leaf anatomy. Skórzynska-Polit et al. [24] subjected runner bean plants to Cd (0.25 µM) stress, noting that younger leaves were thinner, with smaller mesophyll cells and reduced intercellular spaces, whereas in older leaves intercellular spaces were extremely large. More recently, Liza et al. [61] observed minimal changes in the leaf anatomy of chickpea plants under Cd (250–1000 µM) stress, as well as in epidermal or mesophyll tissue characteristics, but reported a reduction in midrib and vascular tissue area and leaf thickness, along with stomata closure.

An investigation conducted by Bouazizi et al. [75] on the Cu-stressed (50 and 75 µM) bean seedling leaves revealed reinforcing of the cell walls of xylem tissues, while the perivascular fiber sclerenchyma appeared to be less developed. Minnocci et al. [76] analyzed the effect of foliar application of Zn fertilizers (150 mg L^−1^) on green beans and concluded that Zn treatment caused an increase in the total lamina thickness, the spongy tissue thickness in particular. In an earlier study, Kasim [77] recorded a decrease in parenchyma cell size and metaxylem vessel diameter in the bean leaf midrib under Zn (600 mg kg^−1^) stress. Shukry and Al-Osaimi [30] observed an increase in lamina, midrib and vascular bundle thickness of bean leaves with increasing Zn concentrations (1, 200, 600 and 1200 mM). Vezza et al. [78] reported a decrease in xylem vessel size in As (25 µM) treated soybean leaves.

## 4. Impacts of Legume–Rhizosphere Microbe Interaction on HM and Phytoremediation

Plants that survive heavy metal stress conditions must adapt/acclimate to evade the severe effects of metal induce toxicity through physiological, biochemical, and molecular mechanisms. Phytoremediation is a sustainable approach to degrading, removing, or immobilizing heavy metals in soil–food crop subsystems through various processes such as degradation (phytodegradation, rhizo-degradation), accumulation (rhizofiltration, phytoextraction), dissipation (phytovolatilization) and immobilization (phytostabilization). Natural nitrogen-fixing plants, the plant-symbiotic microbe interactions are of particular interest in legumes [8]. Microbe-mediated phytoremediation is seen as a potential method of treating heterogeneous pollutants. Studies have reported that the microbes, including bacteria and fungi, play a beneficial role in growth promotion, stress reduction, and degradation [79]. In this section, we outline some recent research advancements in the role of rhizosphere microbe in plant responses to heavy metals and its assisted phytoremediation, which will help more comprehensively understand the mechanism of heavy metal stress tolerance in legume plants and the potential use of plant growth-promoting rhizobacteria (Figure 1).

### 4.1. Role of Rhizospheric Microbes in Plant Responses to HM

Microbes induce various innate plant growth stimulating traits, such as phytohormone synthesis, siderophores, and chelating compounds, and thus play an intermediary role in bioremediation and microbe-mediated removal of various pollutants [79]. Many symbiotic rhizobial strains showing resistance to heavy metals, such as Zn, Pb, and Cu, have been found in legumes growing in polluted regions, such as mine deposits and serpentine soils. These microbes mobilize the pollutants in the rhizosphere region of plants, which are then taken up by plants. They also help plants to resist various environmental stresses. Plants, in turn, release the exudates and enzymes that stimulate biochemical and microbial activities in the adjoining soil, thus supporting bioremediation. Micro-organisms can secrete compounds beneficial for plant growth and thus can promote plant survival in HMs contaminated soils [79]. Responses to HMs are also influenced by hormonal crosstalk between plants and bacteria. It has been suggested that sulphur-amino acid-decomposing bacteria within the rhizosphere of *Helianthus tuberosus* and *Armoracia lapathifolia* growing in Hg-contaminated soil immobilize Hg within soil as HgS, employing sulphur release. Thus, bioavailability of Hg, and consequently root uptake, are reduced [79,80]. Hg immobilization in soil protects plants against toxic concentrations of Hg, allowing revegetation of Hg-contaminated areas. Hence, to achieve removal of the contaminant via plant uptake, Hg-mobilizing bacterial mechanisms have been considered in the context of assisted Hg phytoextraction [80].

Several studies have shown that rhizobacteria can reduce the stress effect of HMs and influence various phytohormones [80] reported that the association of rhizosphere bacteria with HMs hyper-accumulating plants (*Sedum alfredii*) increased metal mobilization by increasing the production of the five (acetic, formic, oxalic, tartaric, and succinic acids) important organic acids. Heavy metal bioavailability can be reduced by sulfate-reducing bacteria (*Desulfovibrio desulfuricans*) by producing insoluble metal sulfide compounds. Metal sulfates have a low solubility, which can cause metal precipitation from soil solutions and reduce metal uptake by plants. The chemical reduction of metals during the processes of sulfate reduction by bacteria leverages protons and enhances the pH of the environment, further reducing metal solubility [81]. Rhizosphere bacteria can excrete organic molecules that chelate Cd^2+^, making it less available to the plants. Gupta and Diwan [82] reported that *Pseudomonas putida*, which secretes extracellular polymeric substances containing carboxyl and phosphate groups to bind Cd^2+^, reduced the bioavailability of Cd to the plant root and bacteria. In *Glycine max*, the co-inoculation of endophytic bacteria with fungi significantly reduced the stress effect of Al and Zn [83]. Bianucci et al. [84] reported that the soybean inoculation with *Bradyrhizobium* sp. *Per 3.61* significantly reduced As toxicity by lowering its translocation and accumulation in edible parts.

It is also reported that nitrogen fixation enhanced plant growth in HMs-contaminated soils. Nitrogen fixation in root nodulation in legume stimulate plant development by increasing phytostabilization of HMs (Cd, Cu, Pb) that reduce metal translocation to aerial parts [85]. Arbuscular mycorrhizae fungi (AMF) were also reported to form a symbiotic relationship with plants, improving nutrient assimilation and stimulating development and growth in heavy metal contaminated soil by increasing access to nutrients, such as P and K. Furthermore, they contribute to the conservation of appropriate soil texture by preventing soil leaching and absorbing heavy metals in their shoots, roots, and leaves and converting them to a non-toxic form [18,86]. Moreover, AMF enhances the host to retain harmful metals in the roots of mycorrhizal plants and restricts transfer to the upper part of the plant [87,88]. The symbiotic relationship between *alfalfa* and AMF reduces the toxic effect of Cd by reducing the translocation and accumulation in the aerial part of plants [89]. Furthermore, in *Medicago truncatula* seedlings under Cu stress, the legume-rhizobium symbiosis regulated gene expression involved in antioxidant responses, phytochelatin (PC) biosynthesis and metallothionein biosynthesis [90].

### 4.2. Rhizosphere Microbe-Assisted Phytoremediation

Studies have indicated that legumes are potentially able to perform phytoremediation and simultaneously boost the nitrogen economy by forming a symbiotic relationship with Rhizobium thereby improving soil fertility and crop productivity [19,91,92]. Arsenic toxicity in soybean alleviated by a symbiotic species of Bradyrhizobium by reducing translocation and accumulation to edible parts of the soybean, avoiding fruit contamination and human poisoning [84] whereas *Sinorhizobium meliloti* increased plant growth and copper tolerance in *Medicago lupulina* [93]. The cool-season model legume *Medicago* has also been analyzed for its phytoremediation ability mainly because of its well-established genetics [37]. Several studies have demonstrated that using *Sinorhizobium (syn. Ensifer) meliloti* and *Sinorhizobium medica* significantly improved nodulation efficiency in *Medicago* plants, which resulted in increased metal bioaccumulation via root nodules thus promoting land restoration and phytostabilization [37,38]. Similarly, *Vicia faba*, *Lupinus luteus*, *Lens culinaris*, *Sulla coronaria*, and recently, *Lablab purpureus* cultivated with consortia of *Bradyrhizobium* sp. 750, *Pseudomonas* sp. Az13, and *Ochrobactrum cytisi* Azn6.2 had significantly improved plant growth and productivity while also more accumulated heavy metals compared to non-inoculated controls [39,40]. Furthermore, plant growth-promoting rhizobacteria (PGPRs), such as *Bacillus, Arthrobacter, Pseudomonas* spp., *Kocuria*, and *Microbacterium*, are shown to play a key role in dissolving various metals for enhanced sequestration in metalliferous soil where metals are tightly bound to the soil through protons and other organic anions by acting as bio surfactants [94]. In iron-deficient environments, PGPR can induce the production of siderophores, which serve as iron chelators, promoting iron availability to both soil micro-organisms and plants. Inoculation of alfalfa (*M. sativa*) with a microbial community containing *Proteus* sp. *DSP1*, *Pseudomonas* sp. *DSP17*, *Ensifer meliloti RhOL6* and *RhOL8* strains enhanced seed germination and early plant growth, and attenuated heavy metal stress by lowering antioxidant enzymes and heavy metal accumulation content, ultimately improving the phyto-stabilization process efficiency [95,96]. Mycorrhizae enhance phytoremediation by trapping heavy metals on fungal mycelium (as a physical barrier) and immobilizing them in the soil through gloaming, limiting their bioavailability, transport, and bioaccumulation in plant tissues [97,98]. It is reported that AMF extra radical mycelium could accumulate 10 to 20 times more Cd per biomass unit than non-mycorrhizal plants in plant roots [99]. According to some recent studies, fungal spores, arbuscules, and vesicles may be involved in the storage of HMs, providing further protection against metal toxicity [96]. Soybean grown in the contaminated soils inoculated with AMF (*Funneliformis mosseae*) were more tolerant in alleviating the toxicity of the metal by retaining the heavy metals in the roots, thereby reducing translocation of Cu, Pb, and Zn in the aerial part of the plant and improving the overall plant productivity [100].

Nowadays through genetic manipulation of their rhizobial microsymbiont, various attempts have been undertaken to increase plant growth in the presence of toxic metal concentrations. One method is to introduce a new heavy-metal resistant gene into the rhizobium. For example, inoculation with wild type *S. medicae* of a genetically engineered *M. truncatula* strain, which expressed a metallothionein gene from *Arabidopsis thaliana* in its roots, resulted in increased Cu tolerance [101]. Similarly, a modified strain was developed by transferring an algal As (III) methyltransferase gene (arsM) to *R. leguminosarum bv. trifolii* that could methylate and volatilize inorganic arsenic in symbiosis with red clover without any negative effect on nitrogen fixation [102]. Additionally, some genes associated with improved legume-Rhizobium symbiosis have been identified [103,104,105]. Therefore, genomic manipulation strategies for improving the rhizobial should be used to increase heavy-metal tolerance. Another option is to develop a phylogenetically-related strain by incorporating large resistance plasmids from a non-symbiotic (but highly resistant), which form a symbiotic metal-sensitive strain. Kong et al., [93] reported that ACC deaminase overproduces *S. meliloti* strain, increased Cu tolerance, and promoted plant growth in the host plant *M. lupulina*. Taken together, heavy metal tolerant-plant growth-promoting rhizobacteria (HMT-PGPR) represent a new eco-friendly ‘green-clean’ technology with tremendous potential for crop growth regulation and polluted soil remediation under heavy metal contamination conditions to increase crop yields and farmer livelihood.

## 5. Conclusions and Future Prospects

Environmental pollution is a key problem that is considered one of the biggest challenges of this century during these rapidly changing environmental conditions, which affect the agricultural productivity of the plant. Legumes that grow in heavy metal-contaminated regions tend to accumulate higher amounts of hazardous metals, leading to contamination of the food chain. If adequate steps are not implemented at the appropriate time, the conditions may significantly worsen. Finding appropriate solutions to protect the environment is an important task to save our environment for future generations. Heavy metals are transported with the transpiration stream in the xylem from the roots to transpiring shoot parts. The anatomical structures of different vegetative parts, such as root, stem, and leaves, are crucial for transporting the HMs into the shoot, their redistribution, and further distribution in aerial plant parts. Anatomical structure plays an important role in heavy metal transport and phyto-volatilization through leaves. Several researchers reported that the inoculation of heavy metal-resistant microbes increases metal uptake. Biotechnological approaches that use plants and micro-organisms (fungi, bacteria, yeast, and microalgae) to detoxify and stabilize HMs have therefore emerged as developing and creative technologies that show increasing potential for restoring HM-contaminated soils. For moderately contaminated soils, biological remediation, such as phytoremediation and PGPR, can be the most environmentally friendly and cost-effective approach. For an effective reclamation, the microbial remediation strategy could be combined with phytoremediation methods such as phytostabilization, phytoextraction, and phytovolatilization. Future research must focus on combining the effect of different microbes on phytoremediation efficiency, such as coupled microbial remediation with organic and inorganic chelating amendments, which must be investigated. Metagenomics approaches and microbial metabolic analysis in conjunction with other omics technologies need to be explored to select promising metal resistance and detoxification genes to determine their specific contribution toward improving key plant attributes, such as quality, yield, shelf life, etc. Moreover, more genomic research such as next-generation sequencing (NGS) technology is required to fully understand the metabolic pathways and the mechanisms involved in microbes’ and plants’ tolerance and detoxification of heavy metals.

## Figures and Tables

**Table 1:** Functions and effects of heavy metals on plant growth and their concentrations in edible parts (mg/kg, f.w.) of legume (Pea) grown in non-polluted and polluted soils.

S.No	Heavy Metals	Functions in Plant	Adverse Effects on Plant	Concentration in Fruits		References
				Non-Polluted	Polluted	
1	Copper (Cu)	Constituent of enzymes;	Disruption of photosynthesis	0.4 ± 0.14	12.6 ± 0.40	[41,42]
		Role in photosynthesis and several physiological processes	Plant growth and reproductive processes			
		Involved in reproductive and in determining yield and quality in crops (disease resistance)	Decreases thylakoid surface area			
2	Nickel (Ni)	Constituent of enzymes	Reduction of: seed germination; protein production and chlorophyll and enzyme production	0.9 ± 0.38	24.7 ± 0.76	[41,42]
		Activation of urease	Accumulation of dry mass			
3	Zinc (Zn)	Constituent of cell membranes;	Reduces nickel toxicity and seed germination	3.0 ± 4.35	25.8 ± 1.53	[41,42]
		Component of a variety of enzymes;	Leaf discoloration called chlorosis			
		DNA transcription;				
		Involved in reproductive phase and in determining yield and quality of crops;				
		Resistance against biotic and abiotic stress;				
		Legume nodulation and nitrogen fixation				
4	Cadmium (Cd)		Decreases seed germination, lipid content, and plant growth	3.0 ± 4.35	25.8 ± 1.53	[41,42,43]
			Disturbs enzyme activities,			
			Inhibits the DNA-mediated transformation in micro-organisms,			
			Interferes in the symbiosis between microbes and plants			
			Increases plant predisposition to fungal invasion			
5	Chromium (Cr)		Causes decrease in enzyme activity and plant growth;	0.4 ± 0.14	12.6 ± 0.40	[41,42,44]
			Produces membrane damage, chlorosis, and root damage			
			Reduces chlorophyll, chlorosis, necrosis;			
6	Lead (Pb)		Inhibits root and shoot growth	8.9 ± 1.76	121.0 ± 1.32	[41,42,45]
			Less biomass production			
			affecting seed germination			
7	Manganese (Mn)	Major contributor to various biological systems: photosynthesis, respiration, and nitrogen assimilation	Inhibiting plant growth: Chlorosis in young leaves, Necrotic dark spots on mature leaves, and crinkled leaves	10.9 ± 1.94	61.6 ± 0.79	[41,42,46]
		Pollen germination, pollen tube growth, root cell elongation and resistance to root pathogens				
8	Cobalt (Co)	Several enzyme and coenzyme operations: Accelerating the nitrogen fixation in legumes	Reduced development and crop yield	1.5 ± 0.00	2.3 ± 0.85	[41,42,47]
		Stem development, coleoptile elongation, bud formation, plant growth enhancement	Chlorosis and necrosis and inhibition of root formation, hindering the nutrient translocation and water uptake			
9	Iron (Fe)	Involved in the synthesis of chlorophyll and maintenance of chloroplast structure and their function	Lack of iron causes yellowing in young leaves due to the plant not being able to produce chlorophyll	583.5 ± 45.27	2098.0 ± 24.02	[41,42,48]
		Helps the plant move oxygen throughout the roots, leaves, and other parts of the plant, producing the green color which showed plant is healthy	Excess iron can produce symptoms of stunted growth and discolored bronzing foliage			

## Data Availability

Not applicable.
